# Rising soil temperature in China and its potential ecological impact

**DOI:** 10.1038/srep35530

**Published:** 2016-10-21

**Authors:** Hui Zhang, Enli Wang, Daowei Zhou, Zhongkui Luo, Zhengxiang Zhang

**Affiliations:** 1 Northeast Institute of Geography and Agroecology, Chinese Academy of Sciences, Changchun 130102, China; 2 University of Chinese Academy of Science, Beijing 100049, China; 3 CSIRO Agriculture, GPO Box 1666, Canberra, ACT 2601, Australia; 4 School of Geographical Sciences, Northeast Normal University, Changchun 130024, China

## Abstract

Global warming influences a series of ecological processes and ecosystems’ stability. Although comprehensive studies have been done to investigate responses of various ecosystem processes to rising air temperatures, less is known about changes in soil temperatures and their impact on below-ground processes, particularly in deep layers. Herein, we used 50 y of temperature data (1962–2011) from 360 sites in China to assess spatio-temporal changes in soil temperatures from the surface to a depth of 3.20 m. We determined, apparently for the first time, that soil surface temperature increased 31% more than air temperature, potentially leading to more carbon release to the atmosphere than predicted. Annual mean surface temperature increased by 2.07–4.04 and 0.66–2.21 °C in northern and southern China, respectively, with the greatest in winter. Warming occurred as deep as 3.20 m. The soil temperature rise was predicted to have increased soil respiration by up to 28%, reinforcing climate warming and extending the potential growing season by up to 20 d across China. However, use of only air temperature to estimate soil temperature changes would underestimate those impacts. In conclusion, these results highlighted the importance of soil warming and of using soil temperature to assess and predict soil processes.

Global warming has been widely debates in scientific, political and social communities over recent decades; furthermore, it has caused changes in various ecological processes and has potential to change ecosystems’ stability^1^,^2^,^3^. In spite of comprehensive studies to investigate responses of various ecosystem processes to rising air temperatures^4^,^5^,^6^,^7^ under global warming, much less is known about changes in soil temperatures and their impact on below-ground processes^8^,^9^,^10^,^11^,^12^,^13^, particularly deep in the soil profile. Temperature change can affect most soil processes, including decomposition and formation of soil organic matter^14^,^15^, mineralisation/immobilization of nutrients (N, P, K, etc.)^16^,^17^, and the subsequent nitrogen transformation (nitrification and denitrification) processes^18^,^19^. Any change in these processes can alter soil carbon and nutrient dynamics, soil fertility and productivity, and greenhouse gas emissions, with effects of ecosystem function and global climate change^15^,^20^,^21^,^22^,^23^. Soil respiration, as a key temperature-dependent process, is biologically mediated; its rate increases exponentially with temperature, roughly following the Q_10_ ≈ 2 rule (rate doubles for every 10 °C increase in temperature) for a wide temperature range^24^,^25^,^26^,^27^,^28^. In addition, several plant processes, including seed germination, seedling emergence, early developmental and growth processes, root extension and growth, are all sensitive to soil temperature change^29^,^30^,^31^. Rising soil temperature directly and indirectly impacts these processes, potentially leading to changes in productivity and stability of ecosystems.

This was the first report of soil temperature changes to a depth of 3.2 m analysed across China, including assessment of potential ecological consequences.

## Results

**Figure 1:**
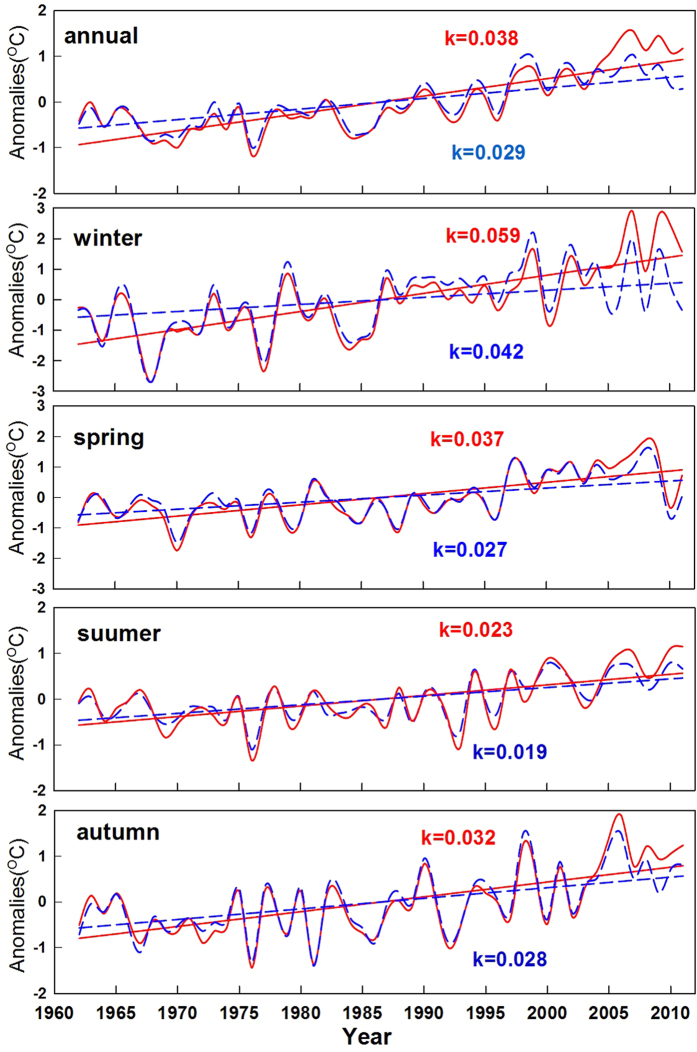
Trends of anomalies of soil surface and air temperatures across China (annual and seasonal scales). Red solid curves and lines represent surface soil temperature, whereas blue dashed curves and lines represent air temperature. Values along the trend lines are rate of change (°C/year).

**Figure 2:**
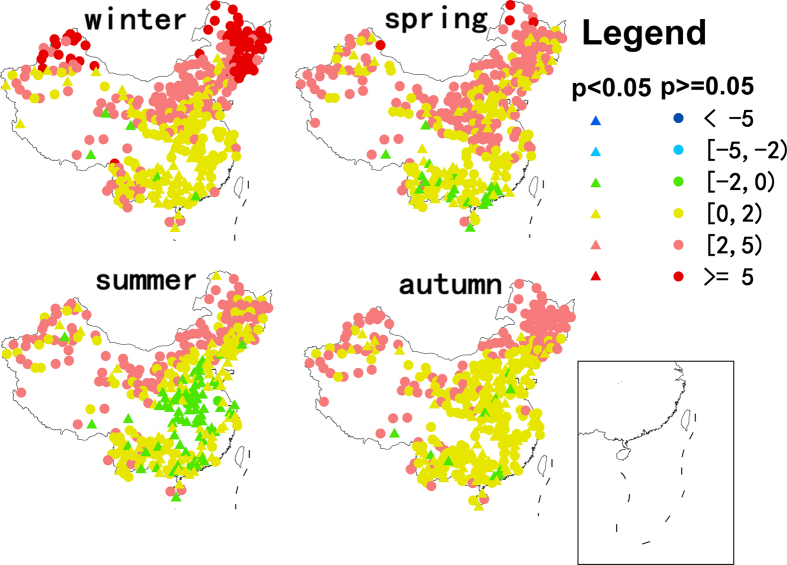
Spatial and seasonal patterns of changes in soil surface temperatures during 1962–2011 (°C) at 360 observational stations in China. The colour of the circles indicates the magnitude of change. Solid circles with point indicate significance level of p < 0.05, whereas solid circles are p > 0.05. Maps in this figure were generated though the ArcGIS 9.3 software provided Environmental Systems Research Institute (http://www.esri.com).

Averaged across all 360 stations, annual surface soil temperature increased by 1.90 °C over the 50-y interval of the study, equivalent to an annual increase of 0.038 °C/y (Fig. 1 and Table 1). This increase was 31% greater than the corresponding change in air temperature (1.45 °C in total, 0.029 °C/y, Table 1). Soil surface warming was greatest in winter (2.97 °C, 0.059 °C/y), followed by that in spring (1.85 °C, 0.037 °C/y) and autumn (1.62 °C, 0.032 °C/y), and was least in summer (1.16 °C, 0.023 °C/y). These seasonal patterns were consistent with air temperature changes, although the increase was greater than air temperature in all seasons (Fig. 1 and Table 1). Soil temperature change also had a clear spatial pattern, with more increases from south to north, and from east to west regions of China. In general, increases were greater in the cooler northern and western regions (Fig. 2).

**Figure 3:**
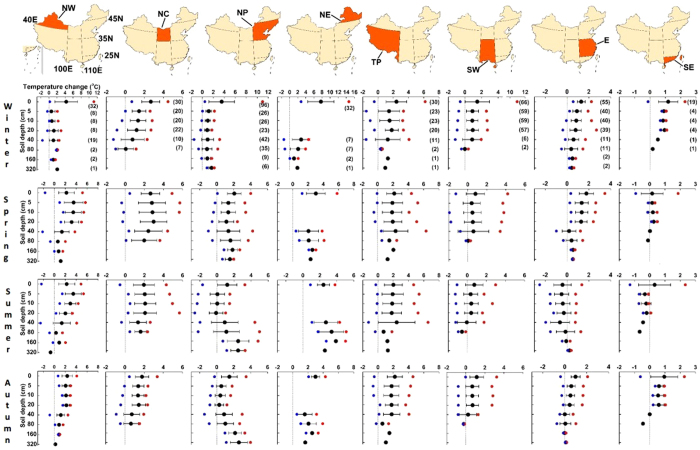
Regional and seasonal patterns of soil temperature change in various soil layers during 1962–2011 in China. The number in brackets is the number of stations where data were used for the calculation in that layer, with black, red, and blue symbols depicting average, maximum, and minimum changes, respectively. Error bars are ±1 SD. The grey line indicates the zero change line. Maps in this figure were generated though the ArcGIS 9.3 software provided Environmental Systems Research Institute (http://www.esri.com).

Overall, warming in soil exceeded the depth of the surface. In all northern regions (Northwest China, North Central China, Northern Plains, and Northeast China) and the Tibet Plateau, soil temperature increased at all depths, from surface to the deepest measurement (3.2 m) in almost all seasons (Fig. 3). Soil warming was greater in shallower layers except in Northern Plains, where more warming occurred in deeper soils, particularly in summer and autumn. Across regions, soil warming at all depths was greatest in cooler regions such as Northwest China, North Central China and Tibetan Plateau (>1.78 °C, 0.036 °C/y), but was negligible in warmer regions like Southwest China, East China, and Southeast China. In the surface soil layer (0.05–0.20 m), national level soil warming was greatest in spring (1.37 °C, 0.027 °C/y) and least in summer (0.63 °C, 0.013 °C/y). In the middle soil layer (0.40–0.80 m), warming was greatest in spring, but lowest in autumn. In the 1.6–3.2 m soil layer, at national level, there was no obvious difference among seasons (~1.60 °C, 0.032 °C/y) except the smaller increase (0.93 °C, 0.019 °C/y) at 1.60 m in winter. This spatiotemporal pattern of change in soil temperatures resulted in more warming in certain soil layers with less warming in others (across regions) as compared to changes in air temperature (Table 1).

**Figure 4:**
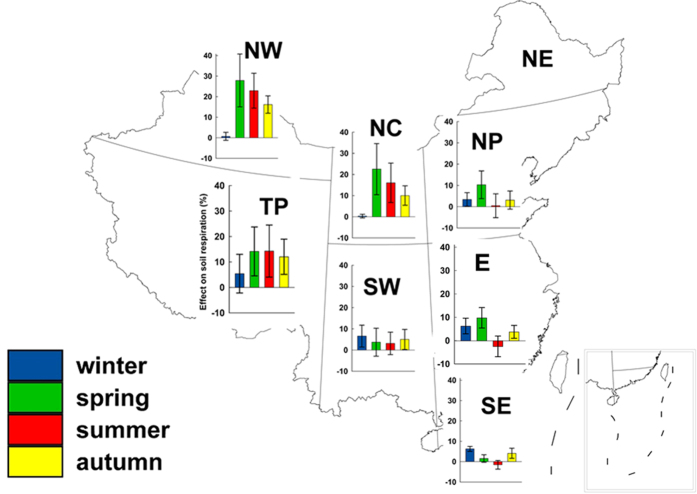
Effect of soil temperature change on soil respiration in four seasons and eight regions in China during 1962–2011. Percentage change of soil respiration calculated using the average value of soil temperature at the 0.05–0.20 m soil layer. The error bars show ± one standard deviation. Map in this figure was generated though the ArcGIS 9.3 software provided Environmental Systems Research Institute (http://www.esri.com).

Over the entire country, on average soil warming was predicted to have increased annual soil respiration by 8.07% (range, 2.59 to 16.92%) in the top 0–0.20 m soil layer (the critical zone of biological activity where most carbon and nutrients are concentrated). Across seasons, the predicted increase in soil respiration was greatest in spring (12.87% on average) and second greatest in summer, except in Southwest China, East China and Southeast China, where the greatest increase occurred in winter (Fig. 4). Soil warming in winter in northern regions had limited effects on soil respiration, because winter temperature in those regions was usually below zero (despite warming), thereby limiting soil biological activity. Across regions, the greatest increase of soil respiration was predicted to have occurred in northern and/or western regions where climate is cooler (Fig. 4). We concluded that the predicted increase in soil respiration over the 50-y interval based on soil temperature increase was much higher than those based on air temperature increases in northwest cooler regions, particularly in spring and summer (up to 16.64% higher; Fig. 4 and Supplementary Fig. 1). We concluded that increased soil respiration enhancement by warming are likely to be underestimated if predictions are based on increases in air versus soil temperature.

**Figure 5:**
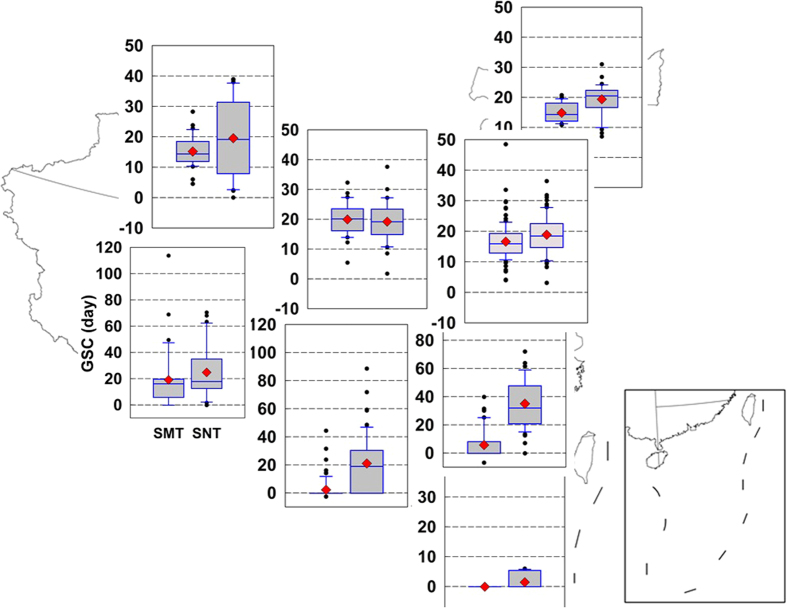
Effects of soil temperature change on durations of potential growing season and frost-free period in four seasons and eight regions in China (1962–2011). Boxplots include the 10, 25, 50, 75, 90 percentiles of the change in duration of soil temperature above 0 °C, calculated from soil mean temperature (SMT) and soil minimum temperature (SNT) respectively. Red diamonds are average and dots are outliers. Map in this figure was generated though the ArcGIS 9.3 software provided Environmental Systems Research Institute (http://www.esri.com).

Based on the change of daily mean surface soil temperature, the estimated date of temperature exceeding 0 °C in spring had advanced, whereas that of temperature decreasing to below 0 °C in autumn had delayed, thereby increasing the duration for surface temperatures exceeding 0 °C (Fig. 5). This duration roughly represents the potential growing season, during which many temperate plants can actively grow. Averaged across China, the potential growing season was extended by approximately 12 d over the 50 y. The greatest extension occurred in North Central China and Tibetan Plateau (20 d), whereas the smallest extension was in East China (approximately 6 d), with no change in Southeast China. The potential growing season extension shared a similar regional pattern with surface soil temperature changes. However, the extension of duration for daily minimum soil surface temperature above 0 °C, roughly representing the frost-free period, was much greater (Fig. 5). Averaged across China, the estimated annual frost-free period was extended by 20 d, mainly resulting from a substantial increase in East China (35 d). The estimated extension with soil temperature changes was 1 and 5 d longer for potential growing season and frost-free period, respectively, as compared to those estimated using air temperature changes (Supplementary Fig. 2). This highlighted the importance of using soil temperatures in ecological studies where soil temperatures are more relevant (e.g., for processes involving soil or early crop growth).

## Discussion

Our results apparently provided the first nationwide images and direct evidence that soil had significantly warmed in China from 1962 to 2011, consistent with previous changes in air temperature^32^,^33^,^34^,^35^, but with a greater warming at soil surface and warming extended to a depth of 3.2 m^34^. We detected a continuous warming trend in soil from 1962 to 2011 in China, without an intermediate cooling period as reported^36^. The predicted increase in soil respiration due to soil warming can be considered an indicator for accelerated decomposition of soil organic matter, increased loss of soil carbon and turnover of soil nutrients. That the extended warming occurred at a depth of 3.2 m (limit of monitoring in this study) indicated a need to investigate its impact on soil processes in deep soil layers, as previous studies usually focused on shallow layers. Soil respiration change over time can be affected by many factors, e.g. vegetative cover change, changes in microbial community etc. There was not enough data to allow analysis of all these influences. However, our results illustrated potential impacts of soil temperature change alone on soil respiration. Hence increased soil respiration needs to be considered together with the changes in the carbon sink in above- and below-ground biomass in calculation of carbon balance in Chinese ecosystems^37^. Soil warming is expected to influence agronomic practices (e.g., sowing date), crop production, and other ecosystem processes such as plant phenology, vegetation shift, niche diversity and ecosystem stability. Such changes evaluated using air or soil temperatures estimated from air temperatures may need to be reviewed and revised, using detailed soil temperature data.

We made substantial efforts to verify the quality of the datasets we used. Consequently, we excluded stations where changes in measurement sites and instruments occurred, and where there were large data gaps. Ultimately only 360 stations were used and there different amounts of data for a given depth in a given region, resulting in some gaps (Fig. 3). Urbanisation has also influenced soil temperatures for the last few decades^32^,^38^. Although its impact is difficult to assess, that it was expected to affect both air and soil temperatures, it should not invalidate our conclusions.

We used a simple approach to estimate potential effects of warming on soil respiration, as one example for changes in soil biological processes. In the absence of soil temperature measurements, most ecological models use air temperature to simulate soil temperature with simple approaches consisting of a delay in time and damping effect with depth (e.g., APSIM^39^). Consequently, estimated soil temperature from such models would have the same long-term temporal changes as those of the air temperature, which would underestimate long-term soil warming and the subsequent impact on ecological processes (e.g., respiration). Thus, our results highlighted the importance to consider differential warming in soil and air. Of course, air and soil temperature changes would have also influenced other ecological processes (e.g., vegetation growth and soil microbial community), leading to more complex influences on soil processes than what our simple approach would suggest. Nonetheless, our results provided substantial insights into potential impacts of soil warming on ecological processes.

Global warming has not only increased air and soil temperatures, but also lead to other changes, including precipitation distribution and vegetation shift. Concurrent changes in temperature and precipitation often cause more frequent drought in many areas^34^, affecting both plant and soil processes, and ecosystems’ responses in general. Increasing winter soil temperatures created more favourable conditions for insects and diseases to survive the winter^8^. In addition, warming also led to faster melting of snow to reduce winter season snowpack and, hence, further affect water availability. All these processes can lead to more complex impacts on ecosystems, although that is beyond the scope of the current study, warranting further research.

## Methods

### Data source and collation

Temperature data were obtained from China Meteorological Administration (CMA) and provincial Meteorological Administration (http://data.cma.cn/data/detail/dataCode/SURF_CLI_CHN_MUL_DAY_V3.0.html), covering 759 national standard meteorological stations across China. These data, which are not yet publically available, included daily maximum, minimum and mean soil temperatures, and monthly maximum, minimum and mean air temperatures from 1962 to 2011. This was apparently the first report of a long-term, nationwide analysis of soil temperature change. All temperature data were measured (CMA standard protocols) in the national CMA weather station network. Soil temperature was measured at eight depths, namely at the surface (0 m), in shallow soils: 0.05, 0.10, and 0.20 m, and in deep soils: 0.40, 0.80, 1.60 and 3.20 m. Surface soil temperature was measured with a glass mercury thermometer, with half buried in soil and the other half exposed in the air. Temperatures in shallow soil were measured using curved glass tube mercury thermometers with the sensing head buried in soil, whereas temperatures in deep soil were measured using glass straight mercury thermometers with the sensor buried at the corresponding depths. All observation sites were bare soil. Temperatures were recorded, on average, four times daily (02:00, 08:00, 14:00 and 20:00) to calculate daily mean soil temperature at eight layers. Furthermore, daily maximum and minimum surface temperatures were recorded by maximum and minimum thermometers.

We eliminated stations that experienced changes in site of measurements, instruments, and severe interruption of continuous soil temperature records (see below). Most weather stations in China began operation in the 1950 s. Because of instrument malfunctions in the earliest years, temperature data before 1962 contained more gaps, especially for soil temperature. Therefore, we used only data from 1962 to 2011. For soil temperature, detailed data quality check involved exclusion of months, with more than one third missing data and exclusion of data outliers. For any month, two thirds of daily records must have been available. This criterion reduced the number of stations (with total 12 mo valid data) to 360. Therefore, daily soil temperature records from 360 stations were used to calculate monthly average soil temperatures. For any month and station, if the calculated average temperature departed from the 50-y average (1962–2011) for that month by three times of the standard deviation from the mean, the value was treated as an outlier, and replaced by the average at the three nearest stations for that month and year. Then average monthly soil temperatures (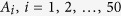) at each site were used. In contrast, monthly air temperatures at the 360 stations were used directly.

### Soil temperature change

Trends for changes in soil temperatures were analysed using the monthly averages across 50 y (for each month, station, region and whole China) together with linear regression and Student’s *t*-test for significance^40^. The rate of change per year was derived as the slope of the linear regression line (*k*), and the total change in 50 y was calculated as *k* × 50.

For each season, i.e. winter (Dec–Feb), spring (Mar–May), summer (Jun–Aug), autumn (Sep–Nov), seasonal average were calculated from those of the three months first, and then seasonal absolute changes were obtained using the same regression process for monthly trend and annual trend.

In order to assess regional patterns of temperature changes, we divided China into eight climatic regions, i.e. Northwest China, North Central China, Northern Plains, Northeast China, Tibetan Plateau, Southwest China, East China, and Southeast China^41^. The eight regions generally reflect climatic and vegetation conditions in China, coinciding roughly with the country’s socioeconomic macro-regions. We averaged changes at individual stations to calculate regional and notational mean changes.

### Potential impact of soil temperature change on soil respiration

We used a simple but commonly adopted approach to estimate potential impacts of soil temperature change on soil respiration^24^,^25^. Soil respiration at a typical temperature can be expressed as:


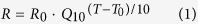


Where *R* and *R*_*0*_ are soil respiration at soil temperatures *T* and *T*_*0*_, respectively, and *Q*_*10*_ is the factor by which respiration is multiplied when temperature increases by 10 °C. We assumed that *Q*_*10*_ was equal to 2, a commonly accepted value^24^.

The relative percentage change of soil respiration (*P*) after soil temperature change of *∆T* (i.e., *T* − *T*_*o*_) can be calculated as:


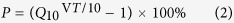


We calculated the *P* with both air (*P*_*air*_) and soil (*P*_*soil*_) temperature changes using *∆T* between 1962 and 2011 estimated based on the absolute change. For this purpose, soil temperature at 0.10 m was used, representing the average temperature in the 0*–*0.20 m soil layer. We further estimated the percentage difference of *P*_*soil*_ relative to *P*_*air*_, i.e.,


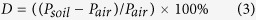


Because both *P*_*air*_ and *P*_*soil*_ were calculated based on actual changes in air and soil temperatures, this calculation enabled us to assess potential bias of soil respiration response predictions based on changes in air temperatures. In many ecological models, soil temperatures were simulated from air temperatures; therefore, changes in the simulated soil temperature would be the same or very similar to those in air temperature. Consequently, such models would underestimate impacts of warming on soil respiration.

### Change in the duration of potential growing season and frost-free period

The duration in days from the time when daily mean surface soil temperature raised to above 0 °C to the time when it decreased below 0 °C was used herein to represent the potential growing season for crops^42^,^43^. We determined this duration for each year and station using the monthly average of daily mean surface soil temperature. The two adjacent months with soil temperatures below and above 0 °C were identified first. Then, linear interpolation of the two monthly temperature values was used to estimate the dates (we called ‘0 °C date’) when the temperature raised and dropped to 0 °C. The use of monthly average of daily temperatures eliminated the need to calculate the moving averages of daily temperatures for determination of the dates when temperatures steadily remained above or below 0 °C. The duration in days between the two ‘0 °C date’ was then calculated for each year and each station. The trend of change in durations was analysed using the same method as applied to soil temperatures. Many eco-biological processes may be activated when the minimum surface soil temperature rises above 0 °C^29^,^31^; therefore, we defined the frost-free period using the change in minimum surface soil temperature, and the same method was used to define potential growing period. We also calculated these two durations using air temperatures for comparison purposes.

## Additional Information

**How to cite this article**: Zhang, H. *et al*. Rising soil temperature in China and its potential ecological impact. *Sci. Rep.* **6**, 35530; doi: 10.1038/srep35530 (2016).

## Supplementary Material

Supplementary Information

## Figures and Tables

**Table 1:** Increase in air and soil temperatures (°C) in China from 1962 to 2011.

	Winter	Spring	Summer	Autumn	Annual								
					NW	NC	NP	NE	TP	SW	E	SE	National
**Air**	2.10^*^	1.33^*^	0.96^*^	1.42^*^	1.60^*^	1.85^*^	1.68^*^	2.08^*^	1.73^*^	0.76^*^	1.18^*^	0.96^*^	1.45^*^
**0** **m**	2.97^*^	1.85^*^	1.16^*^	1.62^*^	2.79^*^	2.26^*^	2.07^*^	4.04^*^	2.21^*^	1.05^*^	0.91^*^	0.66^*^	1.90^*^
**0.05** **m**	1.06^*^	1.35^*^	0.63^*^	0.92^*^	2.42^*^	1.92^*^	0.78^*^	NA	1.78^*^	0.59^*^	0.59^*^	0.34	0.99^*^
**0.10** **m**	1.02^*^	1.38^*^	0.65^*^	0.88^*^	2.31^*^	1.91^*^	0.71^*^	NA	1.76^*^	0.62^*^	0.58^*^	0.35	0.98^*^
**0.20** **m**	1.05^*^	1.38^*^	0.62^*^	0.88^*^	2.16^*^	1.92^*^	0.56	NA	1.84^*^	0.65^*^	0.56^*^	0.40	0.98^*^
**0.40** **m**	1.08^*^	1.45^*^	1.08^*^	0.92^*^	1.27^*^	1.31^*^	1.03^*^	2.30^*^	1.97^*^	0.42	0.04	0.02	1.13^*^
**0.80** **m**	0.81^*^	1.40^*^	1.08^*^	0.85^*^	0.96	1.07^*^	1.15^*^	2.41^*^	0.82	−0.18	0.18	−0.26	1.04^*^
**1.60** **m**	0.93^*^	1.66^*^	2.06^*^	1.74^*^	0.91	NA	1.86^*^	2.53^*^	1.53^*^	NA	0.24	NA	1.60^*^
**3.20** **m**	1.45^*^	1.38^*^	1.68^*^	1.71^*^	0.69	NA	2.09^*^	2.13^*^	1.15^*^	NA	0.32	NA	1.56^*^

^*^P < 0.05.
