# Supplementary material for: Rising soil temperature in China and its potential ecological impact
Source: Sci Rep. 2016 Oct 21;6:35530. doi: 10.1038/srep35530 (PMC5073247; doi:10.1038/srep35530)
Supplement: Supplementary Information [file srep35530-s1.pdf]

Supplementary Information

Rising soil temperature in China and its potential ecological impact

Authors: Hui Zhang[1,2,3], Enli Wang[3]*, Daowei Zhou[1]*, Zhongkui Luo[3], Zhengxiang Zhang[4]

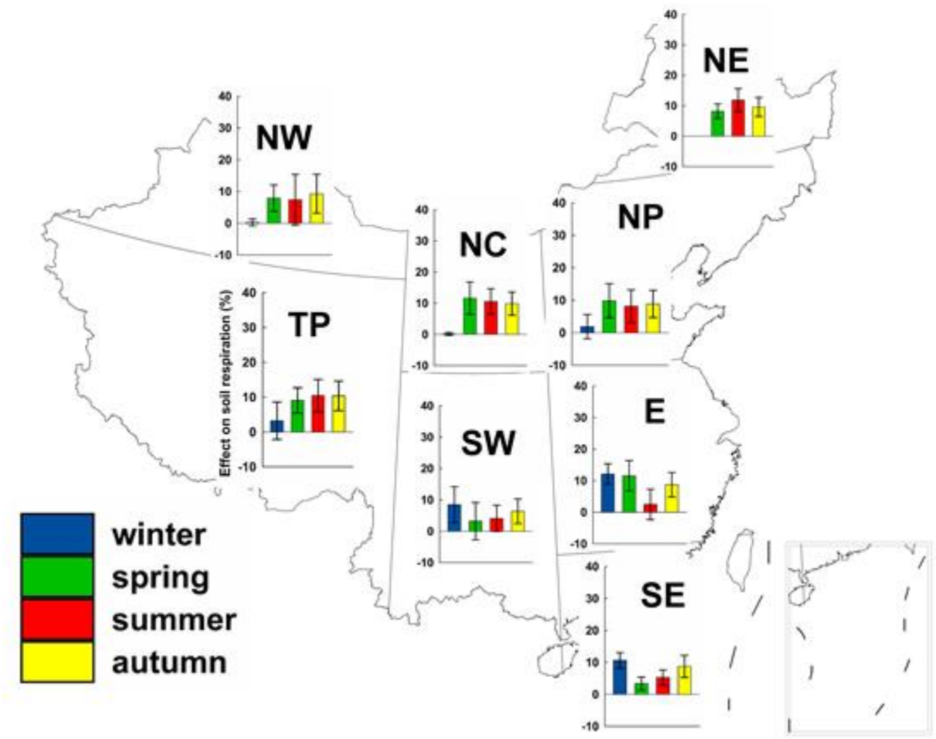

Supplementary Figure 1. The change of estimated soil respiration calculated from change in air temperature across four seasons and eight regions during 1962 – 2011. Map in supplementary figure 1 was generated though the ArcGIS 9.3 software provided Environmental Systems Research Institute (http://www.esri.com).

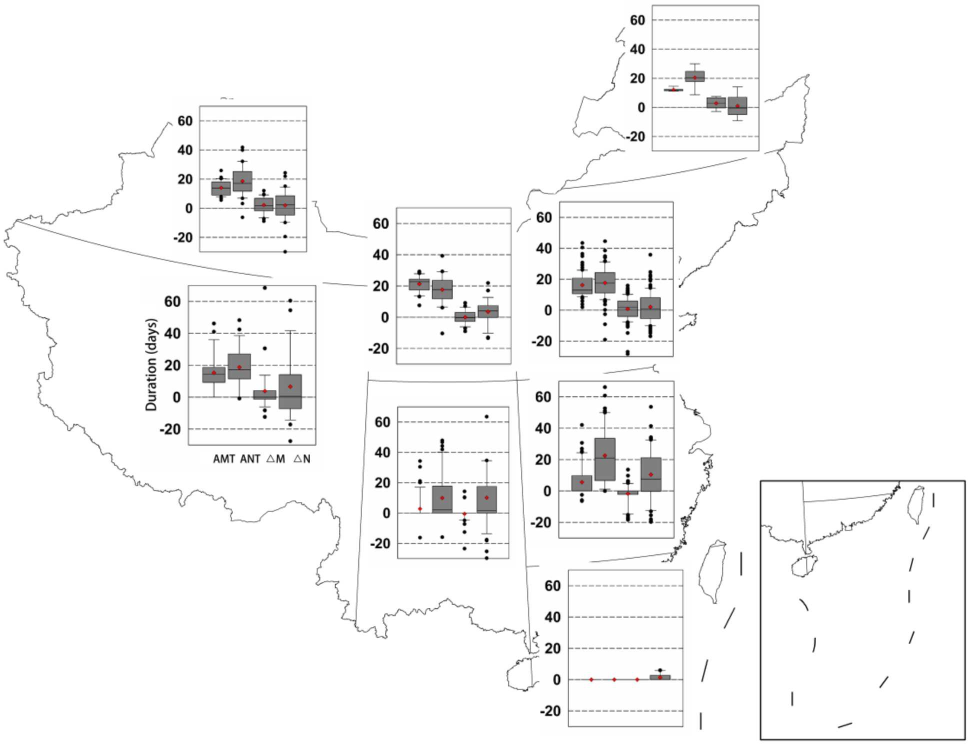

Supplementary Figure 2. Change in potential growing season and frost-free period in 8 regions, 1962 – 2011 estimated using air temperatures and the difference to those estimated with soil temperatures. Boxplots show the 10, 25, 50, 75, 90 percentiles range, calculating from daily mean air temperature (AMT), daily minimum air temperature (ANT). ΔM and ΔN denotes the differences between durations calculated using soil and air temperatures. Dots are extreme data point out of the 10% – 90% data range. Red diamond shows the average. Map in supplementary figure 2 was generated though the ArcGIS 9.3 software provided Environmental Systems Research Institute (http://www.esri.com).
